# Acetylcholinesterase Reactivators (HI-6, Obidoxime, Trimedoxime, K027, K075, K127, K203, K282): Structural Evaluation of Human Serum Albumin Binding and Absorption Kinetics

**DOI:** 10.3390/ijms140816076

**Published:** 2013-08-02

**Authors:** Filip Zemek, Jana Karasova Zdarova, Vendula Sepsova, Kamil Kuca

**Affiliations:** 1 Faculty of Military Health Sciences, University of Defence, Trebesska 1575, Hradec Kralove 500 01, Czech Republic; E-Mails: karasova@pmfhk.cz (J.K.Z.); sepsova@pmfhk.cz (V.S.); kamil.kuca@fnhk.cz (K.K.); 2 Biomedical Research Centre, University Hospital, Sokolska 581, Hradec Kralove 500 05, Czech Republic; 3 Department of Chemistry, Faculty of Science, University of Hradec Kralove, Rokitanskeho 62, Hradec Kralove 500 03, Czech Republic

**Keywords:** acetylcholinesterase, oximes, human serum albumin, pharmacokinetics, reactivator, antidote, nerve agent

## Abstract

Acetylcholinesterase (AChE) reactivators (oximes) are compounds predominantly targeting the active site of the enzyme. Toxic effects of organophosphates nerve agents (OPNAs) are primarily related to their covalent binding to AChE and butyrylcholinesterase (BChE), critical detoxification enzymes in the blood and in the central nervous system (CNS). After exposure to OPNAs, accumulation of acetylcholine (ACh) overstimulates receptors and blocks neuromuscular junction transmission resulting in CNS toxicity. Current efforts at treatments for OPNA exposure are focused on non-quaternary reactivators, monoisonitrosoacetone oximes (MINA), and diacylmonoxime reactivators (DAM). However, so far only quaternary oximes have been approved for use in cases of OPNA intoxication. Five acetylcholinesterase reactivator candidates (K027, K075, K127, K203, K282) are presented here, together with pharmacokinetic data (plasma concentration, human serum albumin binding potency). Pharmacokinetic curves based on intramuscular application of the tested compounds are given, with binding information and an evaluation of structural relationships. Human Serum Albumin (HSA) binding studies have not yet been performed on any acetylcholinesterase reactivators, and correlations between structure, concentration curves and binding are vital for further development. HSA bindings of the tested compounds were 1% (HI-6), 7% (obidoxime), 6% (trimedoxime), and 5%, 10%, 4%, 15%, and 12% for K027, K075, K127, K203, and K282, respectively.

## 1. Introduction

Drug development is challenging work with numerous unknown variables that have to be taken into account, including the detailed pharmacokinetics of novel compounds [1]. Here, we present *in vitro* and *in vivo* determinations of plasma concentration and human serum albumin (HSA) binding potency of newly synthesized acetylcholinesterase (AChE, E.C. 3.1.1.7) reactivators. For newly synthesized compounds, properties of absorption, distribution, metabolism and excretion (“ADME”) are critical for determining the future potency of compounds in clinical practice. Indeed, the “Lipinski rule of 5” quantifies the properties that compounds should possess to be eligible for success [2,3]. This rule postulates that poor absorption or permeation is more likely when there are more than 5 H-bond donors, 10 H-bond acceptors, the molecular weight is greater than 500 and the calculated Log *P* is greater than 5. Lipinsky *et al*. (1997) [2] stated these properties for drug candidates to be used orally, however it can be more or less applied to all drug formulations with appropriate modifications.

Many years before Lipinsky postulated his ADME properties, Sudlow *et al*. (1975) [4] found that HSA interacts with various compounds, and defined two major binding sites responsible in the majority of cases for altering the pharmacokinetic profile of exogenous compounds. The two binding sites were named site I and site II, or Sudlow sites I and II. In addition to these two sites, other less potent binding regions have been identified: e.g., domain III (sub-domain B) for thyroxine, propofol; domain II (sub-domain B) for halothane, ibuprofen; domain I (sub-domain B) for indomethacin, azapropazone. It is important to note that substances can bind to several sites with different affinities [5,6]. Compounds that bind to site I have several structural similarities. Most ligands seem to be dicarboxylic acids and/or have heterocyclic molecules with a negative charge localized in the middle of the molecule. Since structurally different compounds (e.g., bilirubin and warfarin) bind to the same site, this points to the flexibility and adaptability of the Sudlow I site [7–11]. Site II, or Sudlow II, is also known as the indole-benzodiazepine site, according to the ligands with the highest affinity. In general, ligands for this site are often aromatic carboxylic acids with a negatively charged acidic group away from the hydrophobic center, e.g., non-steroidal anti-inflammatory drugs (NSAIDs). Compared to site I, site II is smaller (narrower) and less flexible, which is reflected by the absence of large ligands binding to site II (e.g., bilirubin, hemin, hematin or other porphins) [12–17].

Our work focuses on the pharmacokinetic properties of antidotes for irreversible AChE inhibitors that act directly on the cholinergic system and lead to hyperactivation of the choline system with all accompanying negative symptoms, e.g., bradycardia, hypotension, hypersecretion, bronchoconstriction, GI tract hypermotility, which, if left untreated, lead to death [18]. Furthermore, the simple structure of these inhibitors and their relatively easy and inexpensive synthesis makes them even more dangerous, since it makes them easy to be acquired by terrorist groups and misused against civilian personnel (e.g., sarin, tabun, soman, VX) [19]. However, numerous pesticides (parathion, malathion, methyl parathion, chlorpyrifos, diazinon *etc*.) used every day by farmers around the developing world are also inhibitors of AChE and intoxication is unfortunately very common [20].

**Figure 1:**
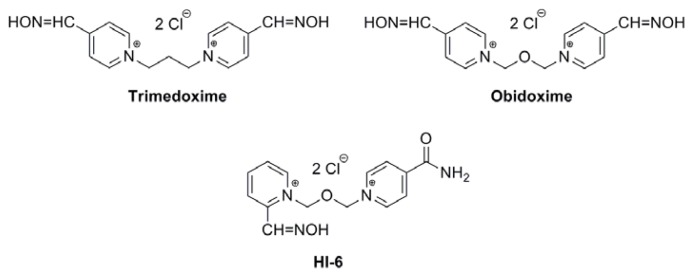
Structures of the standard Acetylcholinesterase (AChE) reactivators used.

**Figure 2:**
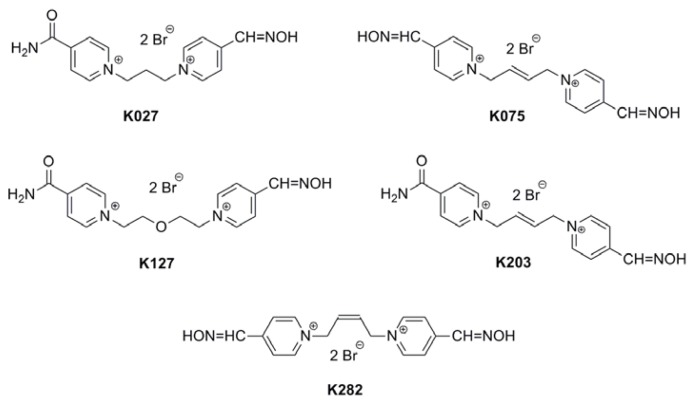
Novel AChE reactivators tested in this study.

In the work presented here, oximes (HI-6, obidoxime, trimedoxime, Figure 1) that are structurally and therapeutically well-defined were used as standards to help correlate the biological behavior of five newly synthetized compounds (K027, K075, K127, K203, K282, Figure 2). Some pharmacokinetic data of HI-6, obidoxime, trimedoxime, K027, and K203 have been published earlier [21–24], but binding to human serum albumin and structural correlations have not been performed before on these compounds, and using a whole set of promising structurally heterogonous AChE reactivators make comparisons more robust. Novel compounds are structural analogues combining features from all three established oximes. *In vitro* tests have confirmed improved reactivation efficacy [25,26], so detailed pharmacokinetic data are needed for further testing [27].

Recently, novel hypotheses and compounds with unique structures have been introduced as potential antidotes against OPNA poisoning (MINA, DAM, non-quaternary oximes, bioscavengers, *etc*.). Nevertheless, more experimental work needs to be conducted to demonstrate the superiority of these new approaches. Currently, quaternary oximes are still the only compounds approved for this use [28–30].

Though based on the known information and structures of the tested compounds significant binding cannot be expected, we here provide essential experimental confirmation.

## 2. Results and Discussion

### 2.1. Tolerability

After *i.m.* application of the tested compounds, no side effects were observed the experimental animals. Moreover, no signs of discomfort such as pain or convulsion of the hind limb muscles were observed during the experimental 240 min or the follow-up period. The follow-up period was conducted 24 and 48 h after the last application. None of the animals showed any convulsions or movement difficulty. Eating and drinking habits were normal.

### 2.2. Plasma Concentrations

Obtained *C*_max_ data from the experiments conducted on male Wister rats are given in Table 1. Kinetic curves where *C*_max_ can be linked to the relevant time interval. HI-6 and trimedoxime exhibit slow elimination from the blood and thereby are present longer in higher concentrations. In contrast, obidoxime has a quick onset and also relatively fast elimination. The kinetic curves of K127, K075 and K282 show even longer elimination periods and relatively stable concentrations over longer periods of time. The kinetic curves of K075 and K282 are quite similar, with both graphs showing a less pronounced curve and missing the characteristic elimination profile, and thus most resemble the profile of trimedoxime. K027 and K203 exhibit a kinetic profile with a relatively well-defined peak determining *C*_max_. These curves can be considered analogous to those of HI-6, and even *C*_max_ values are comparable. Obidoxime seems to have a unique profile with a relatively swift elimination phase and the highest *C*_max_ peak.

### 2.3. HSA Binding

Samples (1 mL) were prepared in triplicates for each compound with one control sample (HSA was substituted by phosphate buffer) and transferred into Centrifree^®^ Ultrafiltration Devices. The acquired filtrate was subsequently analyzed according to previously published methods [31,32]. AUC from the control sample was considered as a reference value of 100%. Filter retention was determined to be below 1%, as declared by the manufacturer. All compounds were tested in triplicates, and the final AUC was the mean of three consecutive measurements and correlated with the measurements of blank samples.

To date there has only been a general consensus that oximes have no significant interactions with HSA and no binding studies had been experimentally performed. Based on the results shown in Table 2 the interaction potential of the tested compounds is low. HI-6, obidoxime, and trimedoxime, which are standardly used in the military, exhibited pharmacologically insignificant binding of 1%, 7%, 6%, respectively. K127 (4%) and K027 (5%) exhibited the same low binding potency as the standards used. However, K075, K203, and K282 had relatively high increases in binding potency of 10%, 15%, and 12%, respectively. Nevertheless, this increase is still insignificant in terms of pharmacological properties.

### 2.4. Discussion

**Figure 3:**
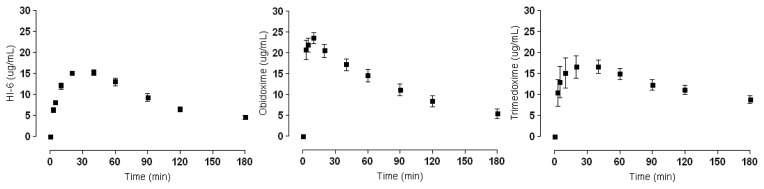
Pharmacokinetic curves of the standards.

All compounds and standards selected for testing are structurally similar, and during *in vitro* screening tests also had high reactivation potency. Based on these preliminary experiments further testing to obtain pharmacokinetic data was performed. We used *in vivo* experiments to acquire relevant kinetic data for these compounds. From Figure 3 it is clear that HI-6 and trimedoxime have an ideal pharmacokinetic profile. However, *C*_max_ is reached only after a relatively long time, which could be a disadvantage when rapid reactivation is needed especially against quickly aging AChE inhibitors (e.g., soman). Nevertheless, HI-6 is the only available oxime so far to show some efficacy against soman intoxication [32]. The *C*_max_ time delay seems to be of importance, since obidoxime reaches *C*_max_ swiftly when compared to the other compounds and has relatively rapid elimination from the plasma. But at the same time obidoxime has demonstrated significant potency against a great number of AChE inhibitors and is part of the military standard antidote kits. Indeed, *C*_max_ values alone cannot be correlated with the therapeutic efficacy of these compounds. Since these compounds serve as antidotes against nerve agents, a clinical trial determining minimum plasma concentrations needed for effective therapy is not feasible. For these reasons, characteristics of these compounds are based predominantly on pre-clinical experimental data.

**Figure 4:**
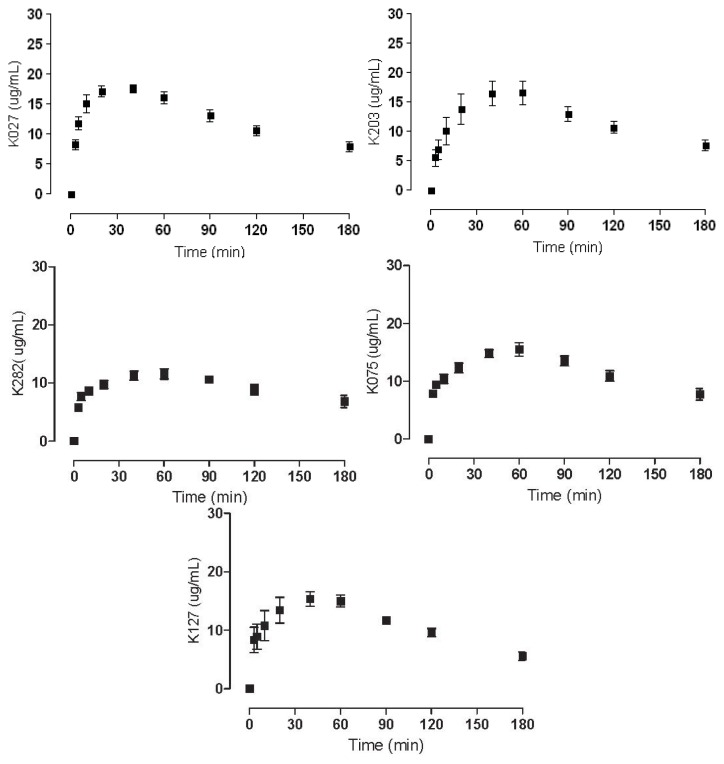
Pharmacokinetic curves of the tested novel oximes.

As is evident from Table 3, the binding to HSA by the studied compounds is insignificant for potential clinical use, not even exceeding 20%. Nonetheless, correlations between binding, structure and pharmacokinetic curves can be drawn. The compounds K075, K203, and K282 have the highest binding potency (Table 3). Furthermore, all three compounds have linking chains between the two pyridine rings of the same length (4C) with a double bond between 2C and 3C. None of the other compounds have similar links (*i.e.*, they either contain an oxygen atom, only 3C, or no double bond). Moreover, the pharmacokinetic curves for compounds K075, K203, and K282 highly resemble each other (Figure 4), with profiles reflecting the possibility that the rise of plasma concentrations is much slower as HSA binds a higher percentage of these compounds, and their *C*_max_ resemble a plateau because HSA is liberating the bound compound. Though these results from the kinetic data are intriguing, crystallographic tests would have to be performed to confirm this hypothesis.

## 3. Experimental Section

### 3.1. Chemicals

Albumin, phosphate buffer saline and acetonitrile super gradient grade G Chromosolve^®^ were purchased from Sigma-Aldrich (Prague branch, Czech Republic). Phosphoric acid (85%) was purchased from Merck (Dermstadt, Germany). HPLC grade water was obtained by a Millipore reverse osmosis system (Goro, Prague, Czech Republic).

### 3.2. Apparatus

The HPLC system used was an Agilent 1260 Infinity Quaternary LC (Agilent Technologies Prague branch, Czech Republic) with a Coulochem II detector-analytical cell model 5011 (ESA, Bedford, MA, USA). Chemstation software (Agilent Technologies Inc., Morges, Switzerland) was used for data acquisition and interpretation.

Protein separation was performed using Centrifree^®^ Ultrafiltration Devices (Millipore, Ireland BV, Tullagreen, Carrightwohill, Country Cork, Ireland). Ultracel regenerated cellulose membrane with a surface area 0.92 cm^2^ designed to retain 99.9% of serum proteins was used.

### 3.3. Sample Preparation

The HSA concentration 45 g/L used in the binding study was derived from the average physiological concentration in a healthy population. The appropriate amount of HSA was dissolved in phosphate buffer saline and left for 15 min in an ultrasound basin to ensure homogenous albumin solution. Samples were prepared by pipetting 900 μL of albumin solution and 100 μL of phosphate buffer saline with each tested substance (plasma concentrations of the tested compounds were derived from pharmacokinetic studies described below) into Eppendorf microtubes and vortexed to ensure a homogenous distribution of the tested compound. Incubation conditions used were 90 min at 37 °C with continuous shaking, followed by sample transfer into Centrifree^®^ Ultrafiltration Devices and centrifugation at 4380 rpm for 90 min, 37 °C. The acquired filtrate was subsequently analyzed on the HPLC system by the method described further below.

### 3.4. Calibration

Calibration curves were established for all measured compounds in the concentration range of 100, 75, 50, 25, 12.5 6.25 and 0 μg/L. All samples were prepared in triplicates and in rat plasma using exactly the same procedure as samples from the actual experiments. The rat plasma was spiked with known concentrations of measured compounds and analyzed the same day. At the end of measurements calibration was repeated to test the precision of the method.

### 3.5. Pharmacokinetic Studies

Selected plasma concentrations of tested compounds were previously determined from pharmacokinetic studies performed on male Wister rats (body weight 230 ± 15 g; Anlab Inc. Prague, Czech Republic). All tested animals were kept in the Vivarium at the Faculty of Military Health Sciences, Hradec Kralove at constant temperature (22 ± 2 °C), humidity (55% ± 6%), and regulated 12 h light-dark cycles. Standard laboratory food and tap water were available ad libitum. The experiment was performed under the supervision of the Ethical Committee of the Faculty of Military Health Sciences, University of Defence, Hradec Kralove, Czech Republic.

All animals were left to adapt for seven days in their new surrounds to eliminate the stress factor. Tested compounds were injected intramuscularly into the hind limb. Applied doses were calculated as 5% of LD_50_[31,32] (tested compounds and doses applied are listed in Table 3). Before application, compounds were dissolved in a saline solution (0.9% *w*/*v* NaCl) of 0.1 mL/100 g of animal weight. Animals were narcotized by an intraperitoneal injection of pentobarbital (50 mg/kg). Cannulation of the arteria carotis was used for blood withdrawals; loss of blood was compensated with saline solution (300 μL) via a cannula in the vena jugularis. Each blood sample was taken just before the subsequent administration in the following time intervals: 3, 5, 10, 20, 30, 40, 60, 90, 120 and 180 min after injection (*N* = 7, seven animals for each compound). All animals survived. Blood samples were centrifuged at 10,000 rpm for 15 min, 10 °C (Universal 320R, Hettich, Germany. The plasma was immediately stored at −80 °C until HPLC analysis.

### 3.6. HPLC Analysis

Before HPLC analysis, plasma samples underwent acetonitrile deproteinization (1:4; plasma/acetonitrile) followed by centrifugation at 14,000 rpm for 15 min, 10 °C. All samples were analyzed in triplicates. All analyses were done on a LiChrospher^®^ 60, 250 × 4.6 (5 μm) analytical column with a 4 × 4 guard column (RP-select B, Merck, Damstadt, Germany). The mobile phase was used for all compounds with slight changes to the pH and the ratio of acetonitrile/purified water or in the concentration of octane sulfonic acid-sodium salt. The composition of the mobile phase used was as follows 20:80 (*v*/*v*) acetonitrile/aqua purificata with octane sulfonic acid sodium salt (6 mM). The pH was adjusted with phosphoric acid (H_3_PO_4_) [31].

The analytical cell and guard cell had voltage set at +350 mV, +650 mV and 1000 mV, respectively. The detector gain was set at 2 μA and all data were acquired at conditioned room temperature (22 °C).

GraphPad Prism, version 5.0 (GraphPad Software, San Diego, CA, USA) was used for statistical analysis.

## 4. Conclusions

Structure influences the pharmacokinetic properties of AChE reactivators and should be considered when designing novel compounds. Naturally, efficacy is the most important characteristic but the behavior of the compounds in the plasma is just as important. Binding to HSA even at lower concentrations can alter pharmacokinetic curves, delay the onset of *C*_max_ and prolong the elimination interval. It seems that a double bond in the linking chain has the potential to increase the binding affinity towards HSA and change the pharmacokinetic profile.

## Figures and Tables

**Table 1:** *C*_max_ of *i.m.* injected AChE reactivators.

AChE Reactivators	Plasma Concentration (*C*_max_ μg/mL)
HI-6	15.26 ± 1.71
Obidoxime	22.76 ± 4.28
Trimedoxime	16.64 ± 4.25
K027	17.61 ± 1.60
K075	15.50 ± 2.82
K127	15.35 ± 3.28
K203	16.63 ± 5.29
K282	11.56 ± 2.31

**Table 2:** Binding of selected compounds to Human Serum Albumin (HSA).

AChE Reactivators	Binding (%) a
HI-6	1
Obidoxime	7
Trimedoxime	6
K027	5
K075	10
K127	4
K203	15
K282	12

a results are the mean of three independent measurements.

**Table 3:** List of tested compounds and applied doses.

Tested Substance	Administered Dose *i.m.* (mg/kg)
HI-6	22.23
Obidoxime	22.23
Trimedoxime	22.07
K027	22.07
K075	23.00
K127	24.39
K203	23.00
K282	23.00
